# Regulation of TGF-*β*1-Induced EMT by Autophagy-Dependent Energy Metabolism in Cancer Cells

**DOI:** 10.3390/cancers14194845

**Published:** 2022-10-04

**Authors:** Jin Seok Hwang, Trang Huyen Lai, Mahmoud Ahmed, Trang Minh Pham, Omar Elashkar, Entaz Bahar, Deok Ryong Kim

**Affiliations:** Department of Convergence Medical Science and Biochemistry, Institute of Health Sciences, Gyeongsang National University College of Medicine, Jinju 52727, Korea

**Keywords:** autophagy, EMT, A549 cells, TGF-*β*1, energy metabolism

## Abstract

**Simple Summary:**

The spread of cancer to other parts of the body is the primary cause of death in most patients. Autophagy is an intracellular maintenance process. Its role in initiating the spread of cancer is still debated. Here, we examined the connection between autophagy and the initial transition of lung cancer cells into the metastatic phenotype, capable of migrating to distant parts of the body. Repressing autophagy reduced the amount of the proteins responsible for this transition but not the expression of their corresponding genes. Therefore, autophagy may regulate the translation of these proteins. We further showed that autophagy involves in regulation of energy mediators, necessary for protein translation. Blocking autophagy and the resulting drop in energy levels diminishes the proteins necessary for initiating metastasis, which represents a potential target of cancer therapy.

**Abstract:**

Metastasis is associated with poor prognosis and is the major cause of death in cancer patients. The epithelial to mesenchymal transition (EMT) is essential for cancer cells to acquire a highly migratory phenotype. Metabolic reprogramming is required to meet the energy demands during this process. Recent studies have indicated that autophagy is involved in EMT, during which cancer cells depend on autophagy activation for survival. However, accumulating evidence indicates that autophagy’s involvement in cancer is context-dependent, acting as either promoter or inhibitor. In this study, we investigated the role of autophagy in supplying energy to support EMT. We induced EMT in Non-small cell lung cancer A549 cells using TGF-β1 with and without autophagy inhibition. Suppression of autophagy activity by knocking down of *BECN1* or chloroquine (CQ) treatment inhibited mesenchymal protein expression. Interestingly, TGF-β1 promoted the transcription of target mRNAs, *SNAI1*, *VIM*, and *CDH2*, regardless of autophagy status. The imbalance between protein and mRNA levels indicated the possibility of autophagy-dependent translational regulation. Since protein synthesis consumes large amounts of energy, it is tightly regulated via various cellular signaling pathways such as AMPK and mTOR. Our investigation showed inhibition of autophagy decreased ATP production from OXPHOS and led to the suppression of mRNA translation by phosphorylation of eukaryotic elongation factor 2 (eEF2). These results suggest that A549 non-small cell lung cancer required autophagy to maintain mitochondrial homeostasis during TGF-β1 induced EMT. In conclusion, blocking autophagy decreased energy production and down-regulated proteins synthesis inhibiting TGF-β1 induced EMT.

## 1. Introduction

Lung cancer patients remain asymptomatic for a long time and are often diagnosed in advanced stages and have a high mortality rate [1,2]. Since metastasis means disseminated cancer cells to distant areas where they form secondary tumors, surgical removal of the lesions is of limited benefit. Therefore, developing new cancer therapies is needed, which requires understanding the underlying mechanisms of metastasis. Epithelial-mesenchymal transition (EMT) was first described during embryonic development, and its role was then extended to wound healing and tumor progression. The importance of EMT in tumor metastasis were well-summarized by [3]. Over the past decades, studies have found that EMT relates not only to metastasis but also immune evasion, chemo-resistance, and even cancer stemness [4].

TGF-β1 is well recognized to promote EMT in epithelial cells [5,6]. It induces the Smad-dependent signaling pathway, which regulates the transcription of epithelial and mesenchymal proteins. As a result of signal transduction, alteration of protein expression affect cell-cell junction, cell morphology, and migration capacities. Analysis of the microenvironment of lung tumors showed increased EMT signal and upregulated expression of TGF-β1 associated genes [7]. Moreover, higher TGF-β1 levels were detected in lung cancer patients [8,9].

According to previous studies, TGF-β1 signaling pathways are associated with autophagy in many biological processes including tumorigenesis [10,11,12,13,14]. In particular, TGF-β1 activate autophagy in non-small cell lung cancer (NSCLC) through either Smad-dependent or Smad-indepedent signaling pathways such as ERK and TAK1 [13,15]. Furthermore, emerging evidence indicates that TGF-β1-mediated autophagy is essential for the EMT process [16,17,18,19]. Nonetheless, the role of autophagy during the TGF-β1-induced EMT remains elusive. Autophagy is a well-conserved process that degrades intracellular components such as proteins, organelles, and pathogens. Although many subtypes of autophagy exist, autophagy consists of three major subtypes: macroautophagy, microautophagy, and chaperone-mediated autophagy. Macroautophagy (hereafter autophagy) is a sequence of processes in which substrates are captured by autophagosomes and degraded by fusing with lysosomes [20]. Inhibiting EMT through manipulating autophagy to suppress metastasis has been repeatedly tested in clinical trials [21]. However, the function of autophagy in EMT has not been elucidated due to its context-dependent characteristics. The complexity of the cellular signaling pathways involved in autophagy partly explains its context-dependence [22]. Therefore, further study of the workings of autophagy in EMT is required.

Cancer cells survive under nutrient restriction owing to autophagy [23], which degrades cellular components to provide energy and building blocks for needed molecules. In addition, the function of autophagy is responsible for the degradation of specific proteins as well as intracellular organelles [24]. Inhibiting autophagy in cancer results in mitochondrial dysfunction [25,26,27]. This study shows altered energy production by modulating autophagy during TGF-β1-induced EMT in A594 cells. These observations indicate that maintaining functional mitochondria for energy production is required for EMT. Inhibiting autophagy suppresses energy production from OXPHOS and inhibits TGF-β1-induced EMT.

## 2. Materials and Methods

### 2.1. Reagents

Antibodies used in this study were purchased as follows: E-cadherin (SC-7870), β-actin (SC-47778), Vimentin (SC-6601), Atg5 (SC-133158), eEF2 (SC-166415), and Beclin-1 (SC-11427) from Santa Cruz Biotechnology (Dallas, TX, USA); pAMPKT172 (2531), AMPK (2532), LC3 (12741), p-eEF2 (2331S), N-cadherin (13116), p-Smad (Ser423/425) (8769) and *SNAI1* (3879) from Cell Signaling Technology (Beverly, MA, USA). Secondary antibodies against rabbit (STAR208P) or mouse (STAR117P) were purchased from Bio-Rad (Hercules, CA, USA). Reagents and drugs utilized in the study: Dulbecco’s modified Eagle’s medium (DMEM, 11995-065), RPMI-1640, fetal bovine serum, Lipofectamine 3000 (11668-500) were purchased from Gibco and Life Technologies (Carlsbad, CA, USA); Protease inhibitor cocktails (78441), Enhanced Chemiluminescence (ECL) detection system (34080), Pierce BCA Protein Assay Kit, RIPA Lysis and Extraction buffer (89900), and TGF-β1 recombinant Human (INV-PHG9214) were from Thermo Scientific (Waltham, MA, USA); Seahorse XF Real-Time ATP Rate Assay Kit (103592-100), Seahorse XF DMEM medium, pH 7.4 (103575-100), Seahorse XF 1.0 M Glucose solution (103577-100), Seahorse XF 100 mM Sodium Pyruvate solution (103578-100), and Seahorse XF 200 mM L-Glutamine solution (103579-100) were from Agilent Technologies (Santa Clara, CA, USA); Mitotracker${}^{\mathrm{TM}}$ Deep red FM (M22426) was purchased from Invitrogen (Waltham, MA, USA); Culture-Insert 3 Well in μ-Dish 35 mm, high for Wound-healing assay were from Ibidi (Grafelfing, Germany); QIazol lysis reagent 200 mL were from Qiagen (Hilden, Germany).

### 2.2. Cell Culture and Transfection

A549 non-small cell lung cancer (NSCLC) cells (from ATCC, #CRM-CCL-185), DU145 prostate cancer (from Korean cell line bank), and MDA-MB-231 breast cancer cells (obtained from Dr. Boothman at UT Southwestern Medical Center) were cultured in DMEM containing 10% FBS. H1299 NSCLC cells (ATCC, #CRL-5803) were cultured in RPMI-1640 medium with 10% FBS. All cells were grown at 37 ${}^{{}^{\circ}}$C in a humidified atmosphere incubator of 95% air and 5% CO${}_{2}$. Cells were transfected with indicated plasmids (sh-*BECN1* (#TRCN0000299864) and sh-eGFP (#SHC005) control, purchased from Sigma-Aldrich) using Lipofectamine 3000 as described by the manufacturer’s instruction (Invitrogen).

### 2.3. Wound-Healing Assay

Cells were seeded and waited for cell attachment in Culture-Insert 3-well dish. At full confluence, culture-Inserts were removed to create two cell-free gaps in which cell migration is visualized. Then, cells in different dishes were treated in various conditions with TGF-β1 to induce EMT, or with 20 μM CQ [28], 100 nM rapamycin [29] to examine the migration condition. The width of the wound area was photographed and measured under an inverted Laboratory Microscope Leica DM IL LED at 0, 24, 48 h after drug treatment. Percentages of wound closure were measured and quantified using NIH ImageJ program (version 1.53e) [30].

### 2.4. Quantification of Cell Elongated Morphology

The axial ratio (AR) was used to measure elongated cellular mophology during the EMT process using following parameters. The axial ratio is defined as:

$$\mathrm{AR}=\frac{a}{b}$$

where *a*, *b* is the length of the major axis and the minor axis, respectively, for the best-fitted ellipse of the cell [31]. At least 200 cells randomly selected from 20 independent areas were used to determine the AR ratio using the NIH ImageJ program (version 1.53e) [30].

### 2.5. Acridine Orange (AO) Staining

Cells were plated in 6-well plates and incubated at 37 ${}^{{}^{\circ}}$C for 24 h. Cells were incubated with AO dye (2.5 mg/mL final concentration) at 37 ${}^{{}^{\circ}}$C for 30 min and washed with PBS. AO-positive acidic vacuoles (AVO) were examined by inverted Laboratory Microscope Leica DM IL LED.

### 2.6. Western Blot Analysis

Cells were collected and lysed with RIPA Lysis and Extraction buffer. The protein concentration of total cell lysates was determined using the BCA Protein Assay Kit according to the manufacturer’s instructions (Thermo Fisher Scientific). Total proteins (20 μg) per lane were used to run on SDS-PAGE gel and transferred to a nitrocellulose membrane using a wet transfer system (Bio-Rad) for 90 min at 80 V. The membrane was blocked for 1 h at room temperature in TBST (10 mM Tris, pH 7.5, 150 mM NaCl, and 0.1% Tween 20) with 5% skim milk. After incubation with primary antibodies overnight at 4 ${}^{{}^{\circ}}$C in TBST with 5% skim milk, the membrane was washed three times in TBST for 10 min each and then incubated with secondary antibodies in TBST for 1 h. The membrane was subsequently washed three times with TBST for 10 min each. Proteins were quantified using the NIH ImageJ program (version 1.53e) [30].

### 2.7. Electron Microscopy Analysis

Cells were fixed in 0.1M PBS with 2.5% glutaraldehyde and then treated in 1% osmium tetroxide buffer. After dehydration in a series of ethanol, the cells were embedded in the EMBed-812 resin (Electron Microscopy Sciences, *14120*). Thin sections (90 nm) were cut on a Reichert Ultracut E microtome and stained with a saturated solution of uranyl acetate and lead citrate. Cells were examined under a TECNAI 12 transmission electron microscope (FEI) at 120 kV.

### 2.8. Determination of ATP Production Pathway

The determination of cellular ATP production was performed using the Real-Time ATP Rate Assay kit in the Seahorse XFe96 analyzer (Agilent Technologies, Inc., Santa Clara, CA, USA). This enables a direct measurement of the intracellular oxygen consumption rate (OCR) and the extracellular acidification rate (ECAR) and thus determines mitochondrial and glycolytic ATP production rates in living cells. A549 cells with different treatments were seeded in a 6-well plate and incubated for 24 h. Cells were seeded in XFe96 microplates at 3 × 10${}^{4}$ per well and incubated for 24 h with or without TGF-β1. The next day, cells were switched to Seahorse XF DMEM medium supplemented with D-glucose (4.5 g/L), 4 mM L-Glutamine, and 1 mM sodium pyruvate for 45 min. Oligomycin and rotenone/ antimycin A were prepared in XF DMEM medium with a final concentration of 1.5 μM and 0.5 μM, respectively, from the Seahorse XF Real-Time ATP Rate Assay kit and were serially injected to measure OCR and ECAR of cells in the XF96 plate. At the end of assays, protein amount was measured by BCA assay for normalization of the results.

### 2.9. Mitochondria Staining

Cells were cultured on coverslips at 60–80% confluence and treated with the indicated drugs for 24 h. After that, cells on the coverslips were fixed with 3.7% formaldehyde in complete DMEM medium for 30 min and subsequently stained with 250 nM mitotracker deep red dye for 30 min. After incubation, the coverslips were washed twice in PBS and then embedded in *VectaMount*AQ mounting medium (Vector Laboratories, Inc., Burlingame, CA, USA) and finally attached on glass slides. All images were obtained under the fluorescence microscope (BX51-DSU; Olympus, Tokyo, Japan).

### 2.10. Quantitative Real-Time PCR

After indicated treatments, total RNAs were extracted from A549 NSLC cells using QIazol lysis reagent (QIAGEN). All performances are following the manual’s description. Total RNAs samples were then treated with DNAase I solution to remove trace amounts of DNA. After that, QuantiNova SYBR Green RT-PCR kit was used to quantify RNA targets in qPCR using SYBR Green I detection. Primers of target genes used in the assay are presented in Table 1. The expression of target genes was quantified in the treated sample relative to the control gene *GAPDH*. RT-PCR data were processed based on $\Delta \Delta C_{t}$ model using pcr R package [32]. Student *t*-text was used to compare the relation expression in target treated groups. *p* values < 0.05 were considered significant. Experiments were performed in at least three replicates.

### 2.11. Statistical Analysis

Each experiment was independently conducted at least three times, and the data were expressed as the mean value (±S.D). Statistical significance between two groups was determined by Student *t*-test using the Prism software (GraphPad Prism, La Jolla, CA, USA). One-way or two-way Analysis of Variance (ANOVA) was used to compare three or more groups, followed by the multiple comparisons Tukey’s test. *p* values < 0.05 were considered significant.

## 3. Results

### 3.1. TGF-β1-Induced EMT Requires Active Autophagy

**Figure 1:**
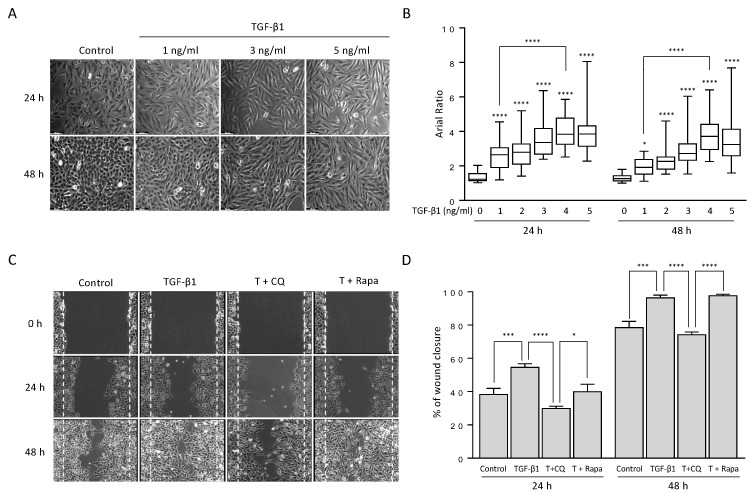
TGF-β1 changed cell morphology from epithelial to mesenchymal-like cells and increased cell migration ability. (**A**) Morphological changes of A549 cells under TGF-β1 treatment. A549 lung cancer cells were treated with the indicated concentration of TGF-β1 for 24 h and 48 h. Bar, 100 μm. (**B**) Box plots for the axial ratio (AR) of A549 cells under TGF-β treatment in a dose-dependent manner. The cell images were transformed to remove the background using ImageJ software. At least 200 cells randomly selected from 20 independent areas were used to determine the AR ratio of straight cells. Data represent the median values with interquartile range in at least three independent experiments. **** *p* < 0.0001 compared to controls were determined by two-way ANOVA followed by Tukey’s test. (**C**) The wound-healing assay. A549 cells were incubated with TGF-β1 4 ng/mL in the presence or absence of 20 μM chloroquine or 100 nM rapamycin for 24 h and 48 h. Bar, 250 μm. The wound edge at 0 h is indicated by white (dotted) lines. (**D**) Quantification of the percentage of wound closure relative to the original wound area. Data indicate the mean values ± s.d of at least three independent experiments. * *p* < 0.05, *** *p* < 0.001, **** *p* < 0.0001 compared to controls were determined by two-way ANOVA followed by Tukey’s test.

To examine the effect of TGF-β1 on non-small cell lung cancer (NSCLC) cells, we treated the A549 cell line with different concentrations of TGF-β1 and assessed the results with phase-contrast light microscopy. As expected, the mophology of A549 cells was transitioned from an epithelial to a mesenchymal phenotype depending on the TGF-β1 concentration (Figure 1A). Indeed, the average axial ratio (AR) gradually increased in a dose-dependent manner (Figure 1B), suggesting that TGF-β1 treatment to A549 cells could induce more fibroblast-like morphology as shown in a previous study [31]. However, the AR ratios exhibted a similar pattern at both 24 h and 48 h although they increased in response to five different TGF-β1 doses (1, 2, 3, 4, and 5 ng/mL), indicating that 24 h after treatment of TGF-β1 is sufficient for the morphological transition from epithelial to mesenchymal. In fact, the relative expression levels of EMT-related proteins were similar at both 24 h and 48 h, as shown and discussed in the next section. In addition, the axial ratio of cells at 1 ng/mL was significantly increased compared to one at 4 ng/mL (Figure 1B). These morphological alterations show that EMT occurs in response to TGF-β1.

TGF-β1-induced EMT in various types of cancer has been associated with activation of autophagy [33,34,35,36,37]. To investigate the involvement of autophagy in these circumstances, we co-treated A549 cells with chloroquine (CQ), an autophagy blocker, or rapamycin, an autophagy inducer, along with TGF-β1. We accessed the migration ability of the cells under co-treatment using wound-healing assay (Figure 1C,D). As expected, A549 cells rapidly close the wound area in the presence of TGF-β1 compared with control. However, up-or down-regulating autophagy resulted in a different ability to close wound areas. Inhibition of autophagy by 20 μM CQ, which blocks fusion of autophagosome and lysosome, significantly decreased the effect of TGF-β1, approximately 25% at 24 h and 48 h. Activation of autophagy by 100 nM rapamycin, which inhibits mTORC1 showed similar results to TGF-β1 at 48 h only. These results suggest that TGF-β1-induced EMT requires autophagy activation.

### 3.2. TGF-β1 Promotes Autophagosome Formation

**Figure 2:**
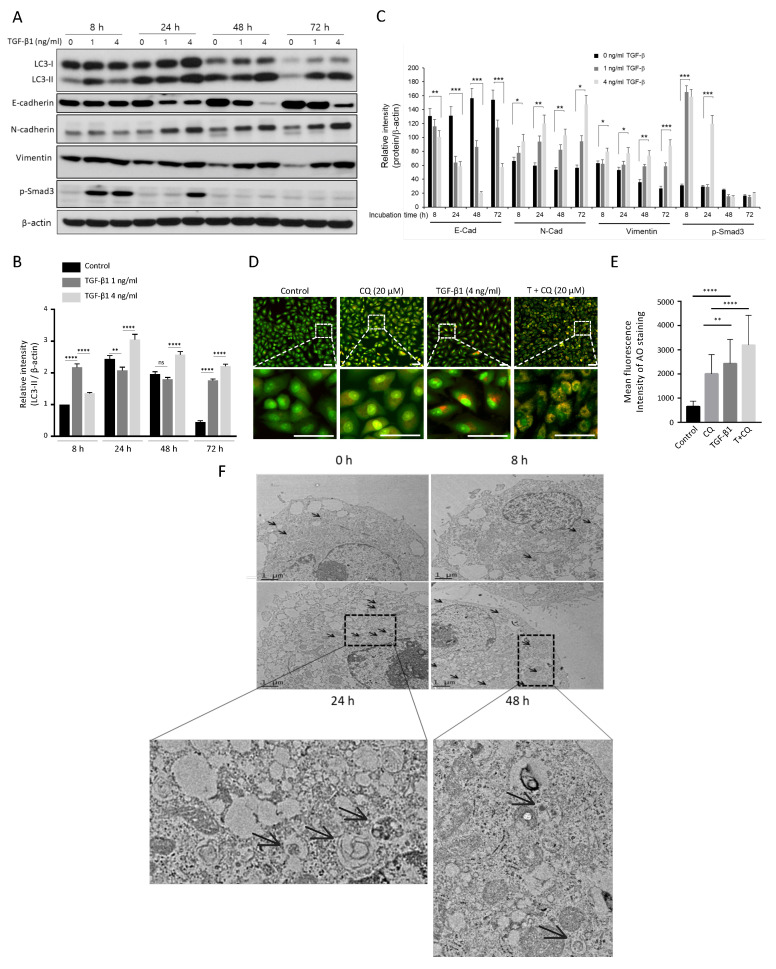
The activation of autophagy is involved in TGF-β1-induced EMT. (**A**) Western blotting. A549 cells were treated with the indicated concentrations of TGF-β1 (0–4 ng/mL) for 8 h, 24 h, 48 h, and 72 h. EMT markers (E-cadherin, N-cadherin, Vimentin, phosphorylated Smad3 (Ser423/425)) and LC3 proteins were detected using their primary antibodies. β-actin was used as a loading control. (**B**) Relative intensity of LC3-II. The relative LC3-II values to LC3-I were quantified using ImageJ software and normalized by β-actin. Data indicate the mean values ± s.d of at least three independent experiments. (**C**) Relative intensity of other EMT markers. The relative values for each EMT marker protein (E-cadherin, N-cadherin, Vimentin, phosphorylated Smad3 (Ser423/425)) were normalized by β-actin. (**D**) AO staining assay. Acridine orange dye stained acidic vehicles of A594 cells incubated with indicated treatments: TGF-β1 4 ng/mL, 20 μM Chloroquine. The bottom images were magnified from the dotted rectangle area. (**E**) The mean fluorescence intensity of AO staining was quantified from at least three independent experiments using the ImageJ program. Scale bar in upper and bottom, 50 μm. (**F**) Transmission electron microscopy (TEM) of A549 cell in treatment with 4 ng/mL of TGF-β1 for 0, 8, 24, 48 h. Autophagosomes and autolysosomes are indicated with black arrows. The bottom images were magnified from a specific area. * *p* < 0.05, ** *p* < 0.01, *** *p* < 0.00, **** *p* < 0.001 compared to control.

The TGF-β1 signaling pathway activates autophagy in different types of cells, including cancer [14,17,38,39,40]. To investigate the TGF-β1 effect on autophagy, we treated A549 cells with TGF-β1 at multiple time points. TGF-β1 treatment mediated expression of EMT-related proteins depending on TGF-β1 concentration and treatment times (Figure 2A,C). Both N-cadherin and Vimentin, mesechymal markers, were highly elevated at 24 h after TGF-β1 treatment, and the expression levels of these proteins maintained similarly up to 72 h (Figure 2C). Conversely, E-cadherin, an epithelial protein, significantly decreased in a dose-dependent manner after 24 h. In addition, the phosphorylated Smad3 (Ser423/425)), a key factor in TGF-β1 signaling, significantly increased at 8 h after TGF-β1 treatment and disappeared after 48 h (Figure 2A,C), suggesting that the early activation of Smad signaling is essential for TGF-β1-induced EMT in this cancer cell as shown previously [8,10,14]. TGF-β1 also altered LC3-II protein level and autophagosome membrane components, indicating the activation of autophagy (Figure 2A). As expected, TGF-β1 treatment dose-dependently increased LC3-II level at all time points (Figure 2B).

Moreover, we stained TGF-β1-treated A549 cells with acridine orange (AO) to visualize its effect on acidic vesicular organelles (AVOs) (Figure 2D). Expanded red areas of cells after TGF-β1 treatment indicate increased autophagic activity. In addition, blocking autophagy by CQ accumulated TGF-β1-stimulated AVO for 24 h. Therefore, the significant increase in the red regions in the case of TGF-β1 and CQ co-treatment compared to CQ alone indicates activation of autophagy by TGF-β1 (Figure 2E). Furthermore, transmission electron microscopy revealed an increased number of autophagosomes as a result of TGF-β1 treatment (Figure 2F). Overall, these results indicate TGF-β1 promotes autophagy activity during EMT progression.

### 3.3. Autophagy Suppression Impairs Mesenchymal Transition and Cell Invasion

**Figure 3:**
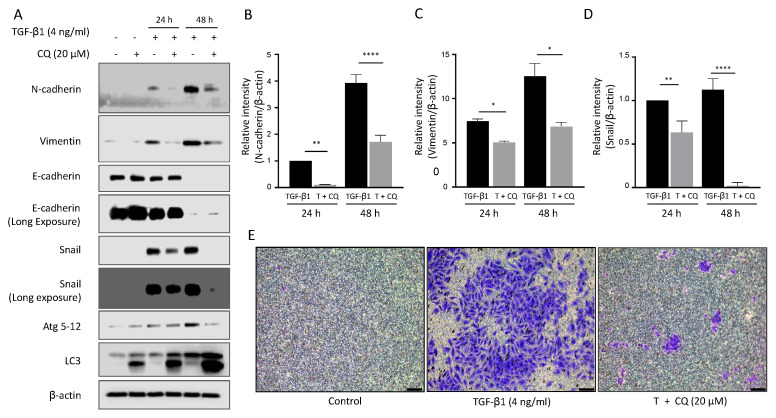
Inhibition of autophagy down-regulates mesenchymal protein expression and invasion ability. (**A**) Western blotting. Protein expression of EMT and autophagy markers in A549 were detected after treatment with TGF-β1 4 ng/mL or 20 μM chloroquine for 24 h and 48 h. (**B**–**D**) Graph representing the relative intensity of N-cadherin and Vimentin, SNAI1 normalized by β-actin. Data represent the mean ± s.d of three independent experiments. **** *p* < 0.0001; ** *p* < 0.01; * *p* < 0.05 *p*-value were evaluated by two-way ANOVA followed by Tukey’s test. (**E**) Invasion assay. A549 cells were placed in the upper chamber of the basement membrane layer under TGF-β1 or TGF-β1 with chloroquine treatment for 24 h. A549 cells that passed the basement membrane at the bottom of the insert chamber were captured by bright-field microscopy. Bar, 100 μM.

To investigate the effect of inhibiting autophagy on EMT, we examined the alteration of EMT protein levels in A549 cells co-treated with TGF-β1 and CQ (Figure 3A). Epithelial cell type A549 maintained E-cadherin expression in control or 20 μM CQ treated cells. TGF-β1 treatment decreased E-cadherin protein level after 48 h. Mesenchymal proteins, N-Cadherin, Vimentin, and SNAI1, were upregulated in response to TGF-β1. Interestingly, treatment with CQ alone did not change E-Cadherin level, but co-treated with TGF-β1 significantly decreased mesenchymal protein levels (Figure 3B,D). As shown before, TGF-β1 increased autophagy protein markers such as LC3-II and Atg5-12. We also stimulated autophagy by incubating A549 with 100 nM rapamycin, which did not promote TGF-β1-induced EMT (Figure S1).

Next, we tested the invasion ability of TGF-β1-treated A549 cells using polycarbonate membrane inserts with or without 20 μM CQ for 24 h (Figure 3E). A549 cells had low invasion ability at basal condition (*left*). As expected based on protein expression change, TGF-β1 significantly increased cell number (*middle*). Inhibiting autophagy by treating 20 μM CQ significantly decreased the cell number. To summarize, TGF-β1 induced EMT and cancer cell invasion ability was restrained by inhibiting autophagy.

### 3.4. Autophagy Modulates Intracellular EMT Proteins through Regulation of Translational Elongation, Independent of Their Transcriptional Activties

**Figure 4:**
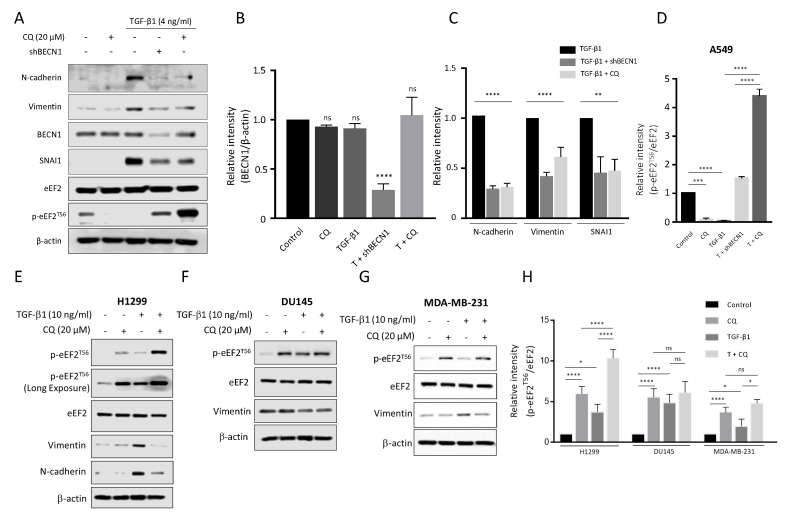
Blockage autophagic flux under TGF-β1 inhibits mesenchymal protein expression and stimulates phosphorylation of eEF2 at T56 in cancer cells. (**A**) Western blotting. A549 cells were transfected with sh*BECN1* or shRNA (eGFP) control plasmid and treated with TGF-β1 4 ng/mL with or without 20 μM chloroquine (CQ) for 24 h. The relative amounts of BECN1 (**B**), other EMT proteins (**C**), and phosphorylated eEF2 at T56 (**D**) in A549 cells were quantified against β-actin using NIH ImageJ software. (**E**–**G**) Western blotting. H1299, DU145, and MDA-MB-231 cells were treated with 10 ng/mL TGF-β1 in the presence or absence of 20 μM chloroquine (CQ). After Western blotting, EMT markers and phosphorylated eEF2 at T56 were detected using their primary antibodies as indicated. (**H**) The relative intensity of p-eEF2 (T56) to total eEF2 in each cancer cell was quantified using ImageJ software. **** *p* < 0.0001, *** *p* < 0.001, ** *p* < 0.01, * *p* < 0.05, ns indicates not significant *p*-value. Statistical test was determined by one-way ANOVA followed by Turkey’s test. Data indicate the mean values ± s.d of at least three independent experiments.

To demonstrate how autophagy regulates mesenchymal protein expression, we disturbed the autophagy activity through CQ or knockdown *BECN1* (Figure 4A). We transfected A549 cells with sh*BECN1*, and its effect was confirmed by reducing the BECN1 protein level at about 74% in comparison to control cells (Figure 4B). Then, the cells were further treated with TGF-β1 4 ng/mL with or without 20 μM CQ for 24 h. We found that N-cadherin, Vimentin, and SNAI1 expressions induced by TGF-β1 treatment were considerably supressed upon inhibition of autophagy with CQ or *BECN1*-knockdown (Figure 4C). We further examined expression of EMT-related proteins in additional cancer cell lines, including MDA-MB-231 (breast), DU145 (prostate), and H1299 (lung) cells treated with 10 ng/mL TGF-β1. Both H1299 and MDA-MB-231 cells exhibited a similar expression of Vimentin upon treatment of TGF-β1 but a decreased expression in the co-treatment of CQ with TGF-β1 (Figure 4E,G) as shown in A549 cells. However, Vimentin expression in DU145 cells, was not significantly affected by either TGF-β1 or co-treatment of TGF-β1 with CQ (Figure 4F), indicating that each cancer cell might differently respond to TGF-β1. Interestingly, inhibition of autophagy under treatment with TGF-β1 in A549 cells exhibited a significantly increased phosphorylation of eukaryotic elongation factor 2 (eEF2) at threonine 56 (T56) (Figure 4A). Indeed, phosphorylation of eEF2 (T56) decreases elongation rate during protein synthesis, which plays an essential role to control the translational step [41]. In particular, decreased eEF2 phosphorylation (T56) in TGF-β1-treated A549 cells was significantly elevated in autophagy-deficient A549 cells by either CQ or *BECN1* knockdown (Figure 4D). This eEF2 phosphorylation (T56) was similarly observed in TGF-β1-treated other cancer cells upon inhibition of the autophagy process by CQ treatment (Figure 4E–H). However, in these cancer cells, the relative levels of p-EF2 at T56 responding to TGF-β1 was different from one shown in A549 cells because they were less sensitive to TGF-β1. we used 10 ng/ml concentration of TGF-β1 rather than 4 ng/mL in A549 cells (Figure 4E–G). These results suggested that inhibition of autophagy could supress protein synthesis in cancer cells during the TGF-β1-induced EMT process.

**Figure 5:**
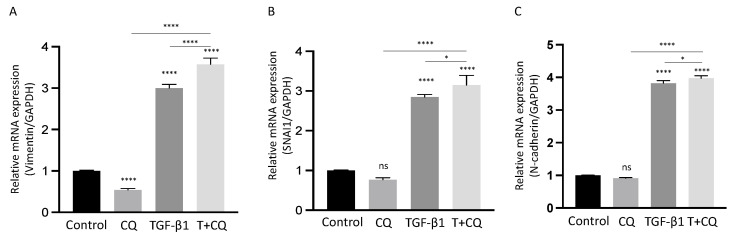
Autophagy inhibition does not suppress transcription of EMT-related genes in TGF-β1-treated A549 cells. (**A**–**C**) The relative mRNA levels of *SNAI1* (**A**), *N-Cadherin* (CDH2) (**B**), *Vimentin* (VIM) (**C**) were determined by Q-PCR in the indicated treatments after 24 h. mRNA levels normalized to *GAPDH*. Data represent the mean ± s.d of three independent experiments. **** *p* < 0.0001, * *p* < 0.05 values were determined by one-way ANOVA with Tukey’s test.

In addition to these results, we examined altered mRNA levels in response to TGF-β1 with or without CQ at 24 h (Figure 5). Interestingly, mRNAs of mesenchymal gene and protein levels were in opposite directions. As shown above, CQ treatment with TGF-β1 decreased mesenchymal protein levels significantly. However, the mRNA levels were similar between TGF-β1 and TGF-β1 with CQ. In addition, Vimentin mRNA showed a higher mRNA level with co-treatment of CQ and TGF-β1 compared with other conditions. These results indicate that, despite autophagy inhibition, TGF-β1 successfully promotes mRNAs transcription, and inhibiting autophagy may modulate EMT through translational regulation of intracellular EMT proteins.

### 3.5. Inhibition of Autophagy Decreases Energy Production from OXPHOX, Leading to Activation of AMPK

**Figure 6:**
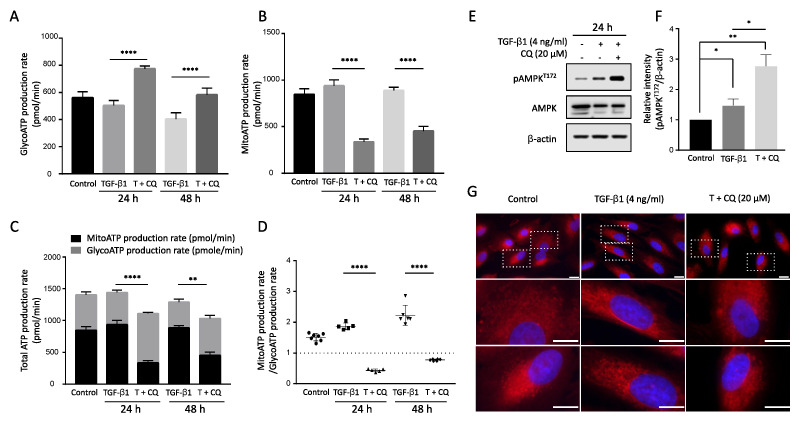
Inhibition of autophagy in TGF-β1 treatment decreased ATP production in mitochondria. Real-time ATP rate assay by Seahorse XF analyzer. A549 cells were incubated with TGF-β1 4 ng/mL with or without 20 μM chloroquine for 24 h and 48 h. The ATP production was distinguished by ATP production rate via (**A**) Glycolysis and (**B**) Mitochondria (OXPHOS). **** *p* < 0.0001 were determined by two-way ANOVA followed by Tukey’s test. (**C**) Total ATP production. The summary of ATP produced from Glycolysis and Mitochondria. (**D**) ATP rate index. The ratio between ATP production from Mitochondria to ATP production by glycolysis. ** *p* < 0.01, **** *p* < 0.0001 were determined by two-way ANOVA with Tukey’s test. (**E**) Western blotting assay of p-AMPK${}^{T172}$ and AMPK protein level in TGF-β1 treatment with or without Chloroquine. (**F**) The relative intensity of p-AMPK${}^{T172}$ to β-actin. (**G**) Mitochondria staining. A549 cells were incubated in the presence of TGF-β1 with or without Chloroquine treatment. Then, mitotracker deep red was used to stain mitochondria. Bar, upper 10 μm and bottom 20 μm. Data represent the mean ± s.d of three independent experiments. * *p* < 0.05 to control was determined by one-way ANOVA followed by Tukey’s test. ns indicates not significant *p*-value.

Protein translation is the most energy-consuming process in the cell and is tightly regulated by the microenvironment [41,42,43,44]. Autophagy is pivotal in maintaining mitochondrial homeostasis by degrading damaged mitochondria. Suppressed autophagy leads to decreased ATP production and even cause apoptosis [45,46,47]. To investigate its effect on energy metabolism, we measured ATP production from cytosolic glycolysis and mitochondrial oxidative phosphorylation (OXPHOS) in A549 cells under TGF-β1 treatment. ATP production via glycolysis (Figure 6A) and OXPHOS (Figure 6B) showed similar levels with TGF-β1 treatment 24 h or 48 h. However, inhibiting autophagy by CQ decreased the total ATP production with opposing outcomes in glycolysis and OXPHOS (Figure 6C). Indeed, the ATP production rate, the ratio of OXPHOS to glycolysis, was gradually increased after TGF-β1 treatment but reversed with the co-treatment of CQ and TGF-β1 (Figure 6D).

Finally, we examined the phosphorylation of AMP-activated protein kinase (AMPK), which acts as a cellular energy sensor during TGF-β1-induced EMT. TGF-β1 increased p-AMPK level, and co-treatment with CQ significantly increased p-AMPK${}^{T172}$ at 24 h (Figure 6E,F). Moreover, we used mitotracker to visualize the effect of inhibiting autophagy via CQ and TGF-β1 treatment on mitochondrial morphology (Figure 6G). Interestingly, cells treated with TGF-β1 had mitochondria with a filiform shape. By contrast, the mitochondria in the cells treated with TGF-β1 and CQ were progressively fragmented, changing to a short punctiform shape. This morphological change correlates with decreased OXPHOS in TGF-β1 treatment, implying autophagy is necessary to maintain mitochondrial homeostasis.

## 4. Discussion

Despite multiple studies focusing on the role of autophagy during EMT, it remains controversial. For instance, autophagy is known to degrade the SNAI1 protein, an essential EMT transcriptional regulator, thus, inhibiting autophagy stimulates EMT [48,49,50]. Alternatively, autophagy promotes EMT by degrading E-Cadherin or increasing TGF-β1 expression [51,52]. These conflicting observations make it difficult to uncover the definitive mechanism of autophagy in EMT for clinical application.

Here, we found that autophagy is required for EMT induction in response to TGF-β1 in the A549 non-small cell lung cancer cell line through regulation of energy homeostasis.

Examining TGF-β1 exposed A549 cells showed increased acidic vesicular organelles or autophagy protein markers that indicate TGF-β1-induced EMT requires autophagy (Figure 1 and Figure 2). In concordance with previous reports, treatment of TGF-β1 induced EMT in A549 cells [18,35,53]. Inhibiting autophagy by CQ significantly delayed mesenchymal protein expression, migration, and invasion (Figure 1, Figure 2 and Figure 3). However, inducing autophagy by rapamycin did not affect EMT (Figure S1). These results contradict our previous report that the inhibition of autophagy promotes EMT, and induction of autophagy attenuates EMT in HeLa, and H1299 [48]. Hela and H1299 cell lines express SNAI1 under basal conditions, and inhibiting autophagy leads to the accumulation of SNAI1 by decreasing its degradation. On the other hand, we failed to detect SNAI1 expression even in the presence of CQ, which indicates that A549 cells expressed a minuscule amount of the protein or did not express it at all (Figure 3). These different characteristics of cancers led to context-dependent results of modulating autophagy.

Although the degradation of intracellular molecules such as protein is considered the main function of autophagy, the role of autophagy has been extended to secretion, organelle homeostasis, and energy metabolism [23,24,54]. Our results showed that suppressing autophagy by CQ or KD-*BECN1* under TGF-β1 decreased mesenchymal protein expression. Although mesenchymal proteins were inhibited upon autophagy inhibition under TGF-β1-induced EMT, the mesenchymal markers mRNAs such as *SNAI1, Vimentin, N-Cadeherin* did not change (Figure 5). These results suggest that inhibiting autophagy triggers intracellular protein regulation rather than controlling the expression of these genes. Modulating autophagy with two drugs, rapamycin, and chloroquine, showed different effects on mesenchymal protein expression (Figure S1). As shown above, autophagy inhibition significantly suppressed mesenchymal protein levels under TGF-β1 treatment. However, inducting autophagy by rapamycin did not affect mesenchymal proteins.

These results indicate autophagy may regulate protein levels via global regulation at the protein synthesis. For instance, if autophagy regulates protein expression by controlling a particular protein, autophagy has to show the opposite result in response to rapamycin or chloroquine.

Furthermore, inhibition of autophagy affects mesenchymal protein expression but does not affect E-cadherin levels. This shows that autophagy is closely related to protein synthesis. Protein synthesis is the most energy-consuming process in the cell and is tightly regulated by energy conditions in response to the microenvironment. Previous studies have revealed that reprogramming energy metabolism under nutrient restriction, or blockage of energy production leads to translational regulation [43,55]. Restricted nutrients or insufficient energy production resulted in an unmatched correlation between mRNA and protein levels [56]. In addition, AMPK, a protein stimulated under low ATP levels, regulates translation via eukaryotic elongation factor 2 kinase (eEF2K). eEF2K is activated by p-AMPK under a high ratio of ATP to ADP, and that phosphorylated eEF2 under nutrient starvation [41]. Other studies have revealed that more energy is required to bring the cells from epithelial to the mesenchymal state [57].

Therefore, we hypothesize that inhibition of autophagy suppresses TGF-β1-induced EMT via translational regulation. The distinct difference between down-regulated mesenchymal protein and up-regulated mRNA levels was shown. In addition, CQ or *BECN1*-knockdown increased phosphorylation of eEF2 specifically under treatment of TGF-β1 not only in A549 but also in other types of cancer cells although this eEF2 phosphorylation at T56 varied among these cancer cells (Figure 4). This differential response of eEF2 phosphorylation to autophagy inhibition among cells might be closely related to the sensitivity of cells to TGF-β1. In fact, A549 lung cancer cells express a high level of TGF-β1 receptors upon expose TGF-β1 [58], and are widely used as a cell model for EMT and cancer metastasis [59,60,61]. Both H1299, a p53-null non-small cell lung cancer (NSCLC), and MDA-MB-231, triple negative breast cancer cell, are less sensitive to TGF-β1 but they undergo substantially the EMT process in response to TGF-β1 [59,62]. Furthermore, the relative phosphorylation of eEF2 at T56 in DU145 prostate cancer cells was less affected by treatment of TGF-β1 even in the presence of CQ and consequently Vimentin was similarly expressed in response to TGF-β1 (Figure 4). To sum, these all results indicate the interplay between autophagy and translational regulation during the cancer progression. Nevertherless, we need to further investigate how these different types of cancer cells respond to TGF-β1 during EMT in the future.

**Figure 7:**
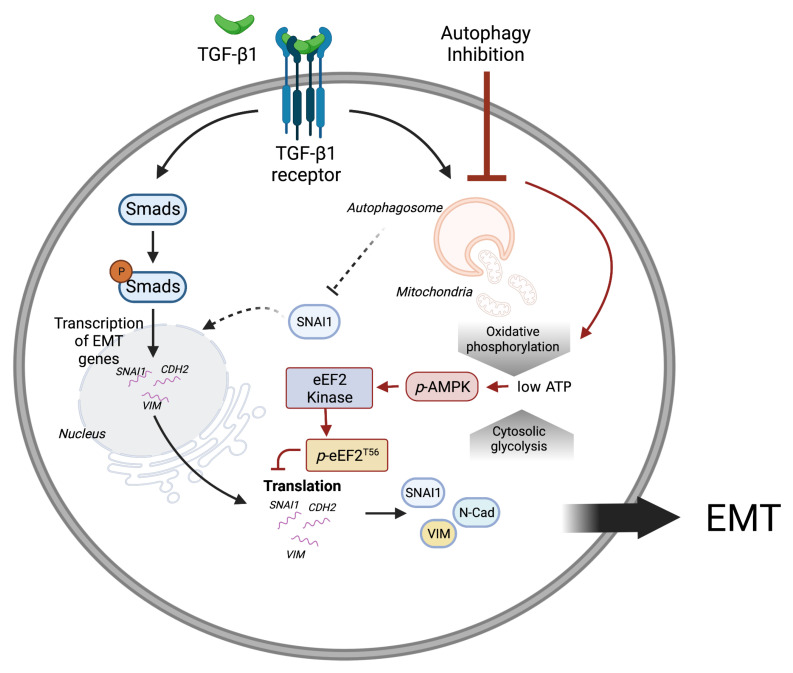
A schematic representation of mitochondrial homeostasis and energy balance by autophagy during TGF-β1-induced EMT. TGF-β1 induces the transcription of EMT proteins through the Smad transcription factors. In addition, TGF-β1 induces autophagy which maintains mitochondrial homeostasis and energy production. Inhibiting autophagy reduces ATP production from oxidative phosphorylation, which consequently suppresses EMT protein translation through AMPK activation. This figure was created with BioRender.com.

Autophagy maintains the homeostasis of cellular organelles, including mitochondria. Previous studies have shown functional relationships between autophagy and mitochondria. Inhibiting autophagy resulted in mitochondrial dysfunction and decreased ATP production [25,26,27]. Our experiments showed that inhibiting autophagy reduced energy production from OXPHOS specifically (Figure 6). Investigate altered energy production revealed that autophagy is vital to maintaining mitochondrial quality, and its inhibition leads to decreased intracellular energy levels. Increased p-AMPK in the combination of TGF-β1 with CQ indicates decreased intracellular energy as shown (Figure 7). Note the graphs show energy production via glycolysis or OXPHOS per minute. In addition, blockage of autophagy causes mitochondrial morphology change. Mitochondria of A549 with TGF-β1 are detected as filament shapes that almost disappear under TGF-β1 with CQ. To date, autophagy has been mainly regarded as the degradation pathway of specific proteins in EMT. However, our findings emphasize the importance of energy metabolism for autophagy-dependent mitochondrial quality control during EMT. This novel aspect of autophagy and EMT relation may explain its context dependence in cancer progression.

## Figures and Tables

**Table 1:** Primer sets used for RT-PCR.

Gene	Forward Primer	Reverse Primer
N-cadherin	5${}^{\prime }$-GACGGTTCGCCATCCAGAC-3${}^{\prime }$	5${}^{\prime }$-TCGATTGGTTTGACCACGG-3${}^{\prime }$
SNAI1	5${}^{\prime }$-CCAGTGCCTCGACCACTATG-3${}^{\prime }$	5${}^{\prime }$-CTGCTGGAAGGTAAACTCTGG-3${}^{\prime }$
Vimentin	5${}^{\prime }$-GGACCAGCTAACCAACGACA-3${}^{\prime }$	5${}^{\prime }$-TCCTCCTGCAATTTCTCCCG-3′
GAPDH	5${}^{\prime }$-TGCACCACCAACTGCTTAGC-3${}^{\prime }$	5${}^{\prime }$-GGCATGGACTGTGGTCATGAG-3${}^{\prime }$

## Data Availability

Not applicable.

## Supplementary Materials

The following supporting information can be downloaded at: https://www.mdpi.com/article/10.3390/cancers14194845/s1.

## Abbreviations

The following abbreviations are used in this manuscript:

AMPK	AMP-activated protein kinase
AVO	Acidic vesicular organelle
CQ	Chloroquine
DMEM	Dulbecco Modified Eagle Medium
ECAR	Extracellular acidification rate
EMT	Epithelial-to-Mesenchymal Transition
FBS	Fetal bovine serum
LC3	Microtubule-associated protein 1A/1B-light chain 3
mTOR	mammalian target of rapamycin
NSCLC	Non-small cell lung cancer
TGF-β1	Transforming growth factor beta 1
OCR	Oxygen consumption rate
OXPHOS	Oxidative phosphorylation
