# Supplementary figures and images for: Regulation of TGF-β1-Induced EMT by Autophagy-Dependent Energy Metabolism in Cancer Cells
Source: Cancers (Basel). 2022 Oct 4;14(19):4845. doi: 10.3390/cancers14194845 (PMC9563415; doi:10.3390/cancers14194845)

|                          |   | 24 h |   |   |   |   | 48 h |   |   |   |   |
|--------------------------|---|------|---|---|---|---|------|---|---|---|---|
| TGF- $\beta$ 1 (4 ng/ml) | - | -    | - | + | + | + | -    | - | + | + | + |
| Chloroquine (20 $\mu$ M) | - | +    | - | - | + | - | +    | - | - | + | - |
| Rapamycin (100 nM)       | - | -    | + | - | - | + | -    | + | - | - | + |

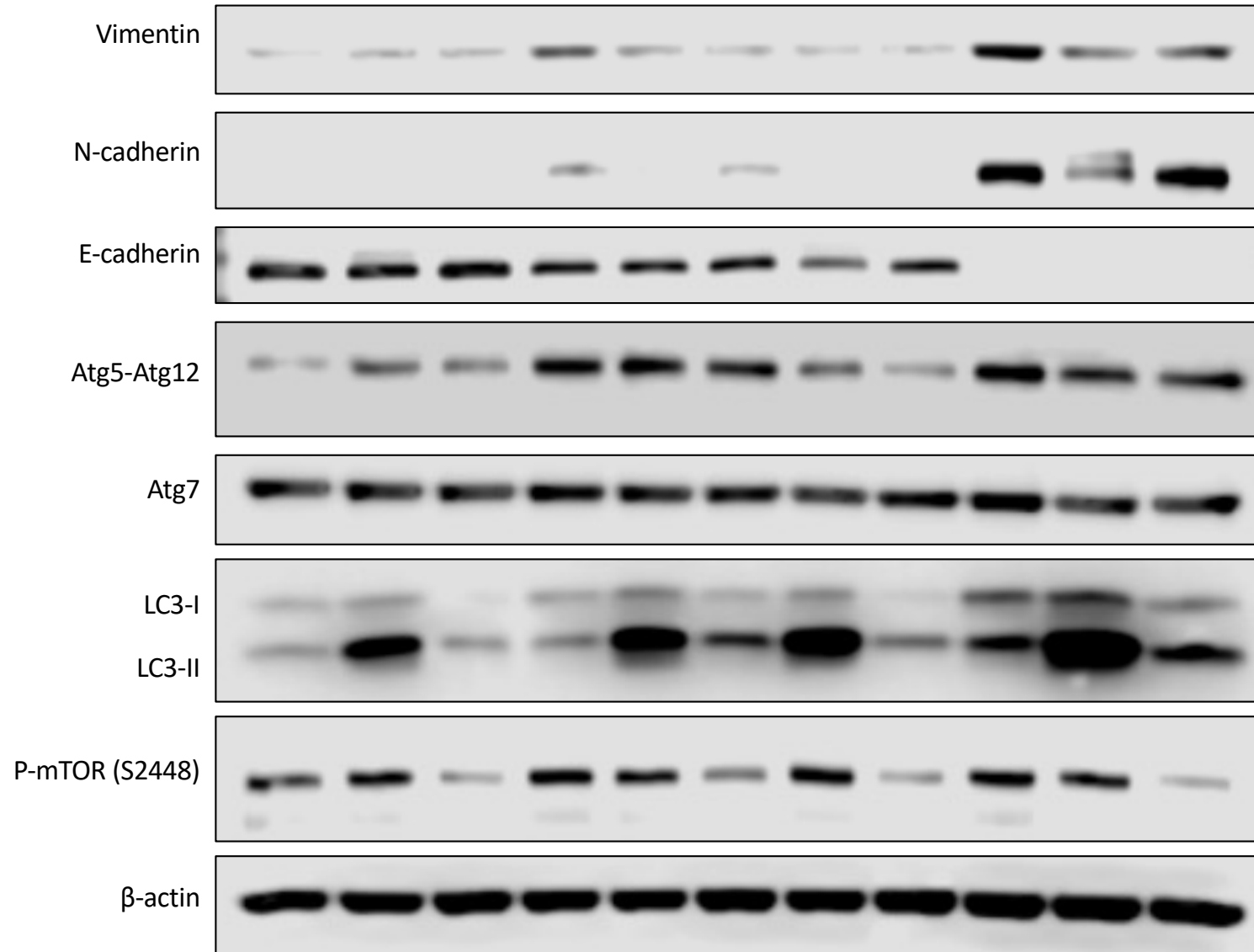

Supplement: Supplementary file 1 [file cancers-14-04845-s001.zip › cancers-1946583-Supplementary Figure S1.pdf]
